# Development of a Model Based on Physical Mechanisms for the Explanation of Drug Release: Application to Diclofenac Release from Polyurethane Films

**DOI:** 10.3390/polym13081230

**Published:** 2021-04-10

**Authors:** Navideh Abbasnezhad, Mohamed Kebdani, Mohammadali Shirinbayan, Stéphane Champmartin, Abbas Tcharkhtchi, Smaine Kouidri, Farid Bakir

**Affiliations:** 1 Arts et Metiers Institute of Technology, CNAM, LIFSE, HESAM University, F-75013 Paris, France; mohamed.kebdani@ensam.eu (M.K.); mohammadali.shirinbayan@ensam.eu (M.S.); stephane.champmartin@ensam.eu (S.C.); smaine.kouidri@ensam.eu (S.K.); farid.bakir@ensam.eu (F.B.); 2 Arts et Metiers Institute of Technology, CNAM, PIMM, HESAM University, F-75013 Paris, France; abbas.tcharkhtchi@ensam.eu

**Keywords:** physical mechanism, modeling, kinetic, drug release

## Abstract

In this study, we present a method for prediction of the drug-release profile based on the physical mechanisms that can intervene in drug release from a drug-carrier. The application presented here incorporates the effects of drug concentration and Reynolds number defining the circulating flow in the testing vein. The experimental data used relate to the release of diclofenac from samples of non-degradable polyurethane subjected to static and continuous flow. This case includes simultaneously three mechanisms: burst-release, diffusion and osmotic pressure, identified beforehand here as being able to contribute to the drug liberation. For this purpose, authors coded the Sequential Quadratic Programming Algorithm to solve the problem of non-linear optimization. The experimental data used to develop the mathematical model obtained from release studies carried out in water solution at 37 °C, for three concentrations of diclofenac and two water flow rates. We discuss the contribution of mechanisms and kinetics by considering two aforementioned parameters and, following that, we obtain the specific-model and compare the calculated results with the experimental results for the reserved cases. The results showed that drug percentage mostly affect the burst release, however flow rate has affected the osmotic release. In addition, release kinetics of all the mechanisms have increased by increasing the values of two considered parameters.

## 1. Introduction

The drug-release profile of drug-delivery systems gives them their efficacy [1]. In-deed, achieving an amount of drug released within a range of the chosen therapeutic window is, for example, an important characteristic sought by researchers [2]. Studies have always tried to design a system with controlled drug release to obtain a desirable release profile [3].

In order to improve the design and achieve the desired release profile from the carriers, it is important to identify the mechanisms interacting in the release system [4,5]. Indeed, to minimize the entropy level of the delivery systems, physical mechanisms occur, which thus leads to the drug-release process [6]. Therefore, physical and chemical mechanisms occurring during the release characterize the release profile.

Usually, drug-release mechanisms from the polymer carriers include diffusion, degradation, swelling, osmosis, etc. Each of these phenomena can lead to a specific release of the drug from the carrier [7,8].

For each mechanism, diverse factors affect the release kinetics [9,10,11]. Among these factors, temperature [12], initial drug load [13], circulating flow [14] pH [15,16], ionic strength, redox and enzymes are, for example, elements whose influence deserves to be studied. In addition, many studies have been conducted to explore the possibilities offered by polymeric biomaterials in regulating drug liberation from stimuli-responsive drug delivery systems. Indeed, drug release could be regulated in response to both human body stimuli [17] and external signals [18]. It is worth mentioning drug release from carriers occurs by one or over one mechanism happening simultaneously or successively. These mechanisms contribute to a part of the global quantity liberated, and each can be affected by the variation of certain factors. Amounts of the drug liberated are limited between 0% and 100% of the global released quantity [19,20]. Therefore, various mechanisms governing drug release need to be adequately investigated. The associated mechanisms are related to the polymer and the drug [21,22]. For many polymers and drugs, the factors influencing the release can vary [23,24]. It is essential to notice the physicochemical properties of the drug, and the matrix can influence the mechanisms. For example, the solubility of particles, permeability and the size of matrix pores. Because of the desirable physical-mechanical properties and biocompatibility [25,26], polyurethanes (PUs) are commonly used in surgical implants and other in vivo products [27]. Based on the physicochemical properties of these carriers, the following mechanisms were identified: Non-trapped drugs in the pores stored on the carrier-surface especially for the hydrophilic drugs induce the burst release [28,29]. However, the trapped drug particles are liberated by other mechanisms like the diffusion, degradation and osmosis [30,31]. The osmosis mechanism is detected by the rapid water absorption or swelling of the non-degradable hydrophilic polymer. Its magnitude is enhanced when the hydrophilic solute is incorporated in the matrix [32,33]. This system is based on osmotic pressure, pushing the solvent through the semi-permeable membrane until there is a balance between the inside and the outside. It is notable that in this case the degradability of the system should be negligible [34]. 

Mathematical modeling is essential in predicting the liberation and kinetic profile by considering the controlling drug release mechanisms. In particular, to facilitate the development of the drug carriers and economizing the time and cost by decreasing the number of experiments in the trial error method. 

Mathematical models for the evaluation of drug-release profiles proceed by solving the equations of the involved physical mechanisms [14,16]; and considering the evolution of kinetics [35,36].

The method used in this study is the sequential quadratic programming algorithm [37], which represents a method that addresses a nonlinear optimization problem. Such an approach is to determine the factors, which reduce a function while maintaining equality and/or inequality conditions on certain other factors. It is a Newtonian algorithm extended to first order optimality conditions.

The method selected is employed to applications of a non-degradable polyurethane matrix loaded with specific doses of the anti-inflammatory hydrophilic drug (diclofenac epolamine). Experimental data used consider several flow rates of the fluid circulating in a bio-reactor simulating in vitro blood-flow in stented arteries [14]. Identified mechanisms for these polymeric carriers are burst-release, diffusion and osmosis. This article discusses the mechanisms contributing to drug release and the material and environmental factors of in vitro assays influencing the kinetic profile. Finally, the dependence of the initial drug concentration and the flow rate on the liberation factors; the burst-release constant k_b_, effective diffusion coefficient D_e,_ and osmotic gradient ∆π were obtained and used for modeling and comparing cases reserved for the verification.

## 2. Materials and Methods

### 2.1. Proposed Method

**Figure 1:**
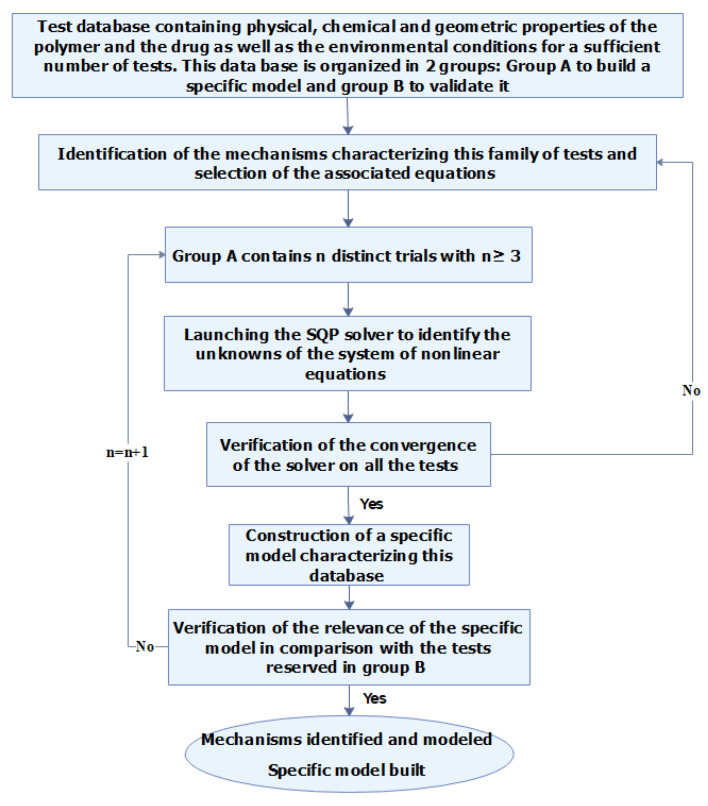
Flowchart of the proposed method.

The method developed in this work is illustrated on the flowchart given in Figure 1. It involves: (i) determining, from a database of well-documented trials, the ratios corresponding to the release mechanisms identified. (ii) By solving a system of non-linear equations, modeling the optimization problem and containing the unknowns for this database. (iii) Developing from these results a specific model simulating the influence of the factors kept. (iv) To validate the model thus constructed on the part of the test-data reserved for this action. 

**Figure 2:**
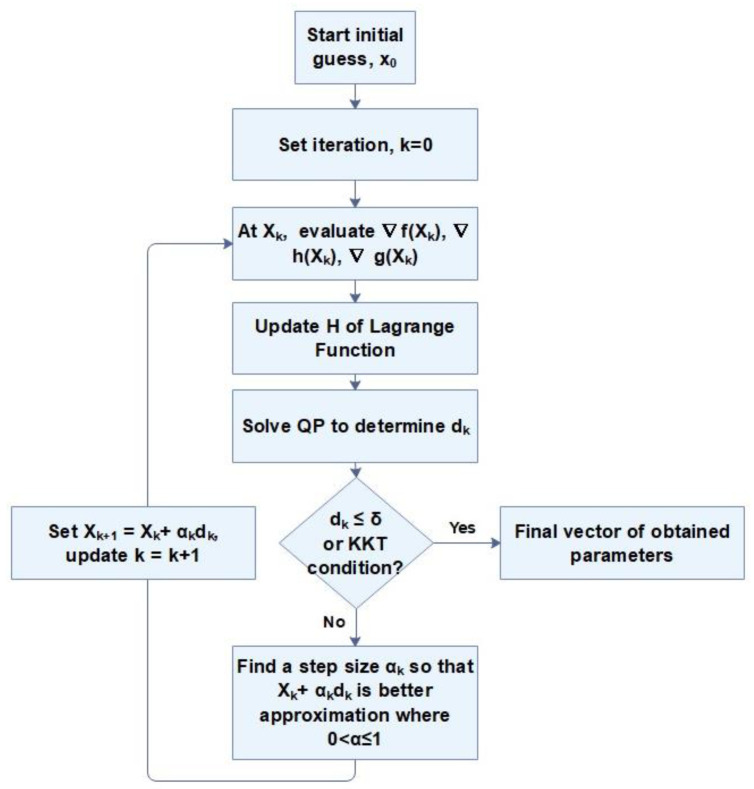
The iterative flowchart of the quadratic programming algorithm (SQP) method [38].

In this section, the developed mathematical model is presented. The proposed method consists of solving a non-linear equations system modeling the all mechanisms identified: burst-release, diffusion, swelling, osmotic pressure, etc. The method operated, known as one of the most effective optimization procedures for solving non-linear optimization problems, is sequential quadratic programming algorithm. Such a problem consists in calculating the factors minimizing a function and simultaneously respecting the imposed constraints. It is a Newtonian algorithm applied to the first order optimality conditions of the system. It finds the solution of nonlinear problems through linear approximations. The quadratic sub-problem (QP) generated is solved following to the steps described in the flowchart bellow (shown in Figure 2). 

The function “f” is differentiable, representing the physical nonlinear model that should adjust to the experimental results. The problem is to find the vector X composed of the unknown parameters minimizing the function f(X). This non-linear optimization problem is subject to the following constraints:

(1) $$h_{i}\left(X\right)=0,\;i=1,2,3,\ldots ,m$$

(2) $$g_{j}\left(X\right)=0,\;j=1,2,3,\ldots ,n$$

The Lagrangian function relative to the current optimization problem defined as:

(3) $$L\left(X,\;\lambda ,\;µ\right)=f\left(X\right)+\lambda h{\left(X\right)}^{T}+µg{\left(X\right)}^{T}$$

where λ and μ are multiplier vectors for equality and inequality constraints, respectively. H in Equation (4) is a Hessian matrix of the Lagrangian function, which calculates the second derivatives of the objective function and constraint functions, h, g. As mentioned before, the quadratic sub-problem (QP) is built by linearization of the constraints expressions, and then the QP can be written as:

(4) $$\mathrm{Min}\;(\nabla f\left(X_{k}{)}^{T}d+1/2\times d^{T}\mathrm{Hf}\left(X_{k}\right)d\right),$$

(5) $$h_{i}\left(X_{k}\right)+\nabla h_{i}{\left(X_{k}\right)}^{T}d=0,\;i=1,2,3,\ldots ,m,$$

(6) $$g_{i}\left(X_{k}\right)+\nabla g_{i}{\left(X_{k}\right)}^{T}d\leq 0,\;j=1,2,3,\ldots ,n$$

Solving Equations (4)–(6) results in a search direction for X noted, d; calculates the acceptable estimates for the Karush–Kuhn–Tucker (KKT) multipliers d, λ, and μ [39,40] updates the matrix H. There are:

$$d\;=\;X\;-\;X_{k},\;\Delta \lambda =\;\lambda -\lambda_{k},\;\mathrm{and}\;\Delta µ=\;µ-{µ}_{k}$$

where Δ symbolize the step of incrementation. H matrix is updated by the Broyden–Fletcher–Goldfarb–Shanno method. The solution converges if the vector, d, is within an accepted tolerance range (δ) of 0.0001, with KKT conditions fully satisfied [39,40]. A step size α is set to ensure the decrease of the objective function [41]. The iterative solution technique described above is repeated until the solution X is obtained. This optimization algorithm is programmed under Matlab software.

The total release profile is consist of different mechanisms contributed in the drug liberation. Therefore, the sum of these mechanisms multiply to the percentage of participation results in the release profile:

(7) $$X=\left(\mu_{1},\;\mu_{2},\;\mu_{3},\;\ldots ,\;\mu_{N}\right),\;\;f\left(X\right)=\;\frac{M_{t}}{M_{\infty }}=\overset{i=N}{\underset{i=1}{\sum }}\mu_{i}\times F_{i}$$

Examples of equations describing mechanisms are given in the case presented in the section below.

### 2.2. Case Study

In this study, we used the experimental results of diclofenac epolamine (DE) release. DE is an anti-inflammatory and a model of the hydrophilic drug (water solubility of 5.55 mg/mL [42]), used in this regard, from the non-degradable polyurethane samples. The samples were prepared with the synthesis of hardener consisting of isocyanate type 4,4-diphenylmethylene diisocyanate (MDI) and resin composed of polyol, dye, and catalyst. The ultimate product consists of a mixture of the resin and the hardener in a ratio of five to two, and certain percentage of the drug (drug mass to the total mass of sample). The samples were cut with the dimensions of 2 × 5 × 30 mm^3^. As the physical stability of the DE in the polyurethane samples were important for considering the mechanisms of release, therefore, it was checked during the samples preparation by microscopic observations. Diclofenac particles were stayed in their initial particle shape, solid-state, after the samples were prepared [14]. These experiments were carried out in vitro on samples loaded by 10%, 15% (for model verification), 20% and 30%, and assessed at four different flow rates: 0, 7.5, 6.5 (for model verification), and 23.5 mL/s. These data have made it possible to observe and differentiate the drug-liberation profiles, in the static and the presence of a continuous flow.

**Figure 3:**
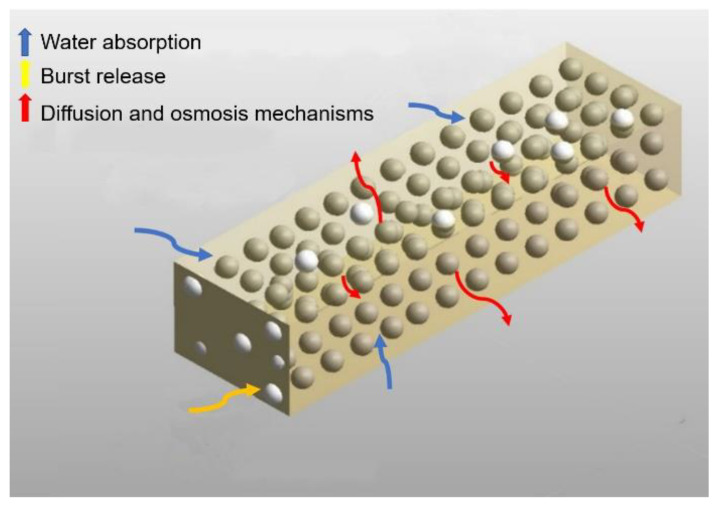
Schematic of the phenomena occurring during the release. Yellow rectangle is the polymer matrix, white spheres are the drug particles on the surface contacting with outside, golden spheres are drug particles inside the polymeric matrix. The arrows show the phenomena of water absorption, burst release and mechanisms of diffusion and osmosis.

Figure 3 shows the schematic of the mechanisms occurring during the release. At first, the fluid transports the drug particles on the surface of the sample; then the water penetrates in the sample and dissolves particles, then the dissolved drugs release by diffusion and/or osmotic pressure.

Equations employed in this case study related to the three phenomena (burst release, diffusion and osmosis) intervene release are grouped by assigning each related coefficients:

(8) $$f\left(X\right)=\;\frac{M_{t}}{M_{\infty }}=\mu_{1}\times F_{1}+\mu_{2}\times F_{2}+\mu_{3}\times F_{3},\;\sum_{i=1}^{i=N}\mu_{i}=1,\;\mathrm{and}\;1\geq \mu_{i}\geq 0$$

where $M_{t}$ represents the quantity of drug released at time t, $M_{\infty }$ is the amount initially loaded,${\;\mu }_{i}$ the contribution of each of the N mechanisms involved and $F_{i}$ the corresponding equations. According to the numerous studies burst release is defined as [43]:

(9) $$\frac{\mathrm{dC}}{\mathrm{dt}}=-k_{b}C$$

where the initial mass of the release at the beginning of the experiment (t = 0) is considered equal to the zero. The model is reformed to the below equation:

(10) $$F_{1}=\frac{M_{t}}{M_{\infty }}=1-\exp\left(-k_{b}t\right)$$

where K_b_ represents initial burst constant and t is the time of release. Diffusion is the period of the release, normally followed by the slow release rate, which is happening through the polymer matrix containing few small pores. It is derived from Fick’s second law [44]:

(11) $$\frac{\partial c}{\partial t}=D\frac{\partial^{2}c}{\partial x^{2}}$$

where D is the drug diffusion coefficient inside the polymer matrix, c corresponds to the concentration of the drug in the polymer network as a function of time t and position x. The initial presumption is that at the beginning of the trial, the drug is dispersed evenly in the film:

$$t\;=\;0,\;c\;=\;c{\;}_{\mathrm{initial}}\;-L\leq x\leq +L$$

where c _initial_ indicates the initial amount of drug in the sample, and L describes the film’s half-thickness. For the resolution of the diffusion mechanism, the hypothesis considers that the product is homogenously distributed throughout the polymeric networks. Moreover, the film surface is very high compared to its thickness (150 mm^2^/2 mm). Consequently, edge effects are insignificant, and the statistical study can be confined to one dimension. Under these conditions, the release kinetics can be defined in a plane sheet for the samples in the shape of the matrix, where it is described as below [20,45]:

(12) $$\frac{M_{t}}{M_{\infty }}=4{\left(\frac{D\;t}{{\pi h}^{2}}\right)}^{2}$$

where π is 3.14 and h is the thickness of the matrix. In theory, this equation is valid for the 60% of the cumulative drug release. It means 0 ≪ release fraction ≪0.6.

It is believed that the drug concentration far from the film’s surface is constant and equivalent to zero since the release medium is well stirred and complete sink conditions are established throughout the experiments [44,46]. Therefore, Equation (12) is completed in the below form:

(13) $$F_{2}=1-\frac{8}{\pi^{2}}\overset{\infty }{\underset{n=1}{\sum }}\frac{1}{n^{2}}\exp\left(-\frac{\pi^{2}n^{2}D_{e}t}{h_{1}^{2}}\right)$$

The other related phenomenon is the osmosis, Equation (8) shows the representative equation for this phenomena [47,48]:

(14) $$\frac{{\mathrm{dM}}_{t}}{\mathrm{dt}}=\left(\frac{A}{h}K^{\prime }\left(\Delta \pi -\Delta P\right)S\right)$$

where A and h are the surface area and thickness of the polymer films (by the hypothesis that the polymeric carrier consist of the porous structure and hydrophilic drug), $K^{\prime }$ is the effect of mechanical permeability (Lp) and reflection coefficient (σ), Δπ represents the osmotic gradient across the film membrane, S is the saturation solubility of the drug in the dissolution medium. According to the thin thickness of the samples and where the accumulation of the water in the samples are not sufficient to induce the hydrostatic pressure and, therefore, hydrostatic pressure inside the system is minimized as expressed by the condition ∆π >> ∆P. Another condition is considering the boundary condition where drug released concentration at time, t = 0 and t = t is zero and $M_{t}$ respectively. Moreover, at t = 0, $\frac{M_{t}}{M_{\infty }}$ equals to zero, therefore, constant value as a result of the integral is equal to zero. Finally, Equation (15) is adapted to the below form:

(15) $$F_{3}=\;\frac{M_{t}}{M_{\infty }}=t\;\frac{A}{h}\;\left(K^{\prime }\Delta \pi S/M_{\infty }\right)$$

The final equation for the release in this system follows as below:

(16) $$\begin{array}{c} \frac{M_{t}}{M_{\infty }}=\left(\mu_{1}\times 1-\exp\left(-k_{b}t\right)\right)+(\mu_{2}\times 1-\frac{8}{\pi^{2}}\overset{\infty }{\underset{n=1}{\sum }}\frac{1}{n^{2}}\exp\left(-\frac{\pi^{2}n^{2}D_{e}t}{h_{1}^{2}}\right))+(\mu_{3}\times t\;\frac{A}{h}\;\left(K^{\prime }\Delta \pi S/M_{\infty }\right)\\ \mu_{1}+\mu_{2}+\mu_{3}=1,\;\&\;1\geq \mu_{1\&2\&\;3}\geq 0 \end{array}$$

where the values of $\mu_{1}$, $\mu_{2}{\;\mathrm{and}\;\mu }_{3}$ represent successively the proportions of the contribution of the phenomena of burst-release, diffusion, and osmotic pressure. The other unknown factors are k_b_, D_e_, Δπ.

The differences of the pressure for different flow rates of the flow in the porous membrane is calculated by the Poiseuille equation adapted for the porous media, with the conditions that the fluid is laminar and incompressible [49,50].

(17) $$\Delta P=\frac{8\cdot \eta \cdot \delta \cdot {\;J}_{v}}{n\cdot \pi \cdot a^{4}}$$

δ is the thickness of the membrane (m), $J_{v}$ is the volumetric flux (m^3^/m^2^·s) and η is the fluid viscosity (Pa·s), a is the radius of the pores (m), that the fluid flows in and n is the density of the pores in the membrane, and:

(18) $$J_{v}=\Delta P\cdot L_{p}$$

From here the value of the L_p_ is obtained as [49]:

(19) $$L_{p}=\frac{{n\pi a}^{4}}{8\eta \delta }$$

Moreover, the reflection coefficient is considered as [49,51]:

(20) $$\sigma =\frac{\pi_{\mathrm{eff}}}{\mathrm{iRTC}}\;={\left(1-{\left(1-\emptyset \right)}^{2}\right)}^{2}$$

where $\pi_{\mathrm{eff}}$ the detected osmotic pressure and iRTC is the theorical osmotic pressure that would be noticed in the perfectly semipermeable membrane. ∅ is the ratio of the radius of the solute particles to the pore, where it is calculated by the SEM images.

By the values obtained from the above equations, the specific model is elaborated and compared with the results of the experiments. The two indicators, root mean squared error (R_MSE_) and R-squared, are used to assess the performance of this model.

(21) $$R_{MSE}=\;(\frac{1}{n}\;\sum \;(y_{\mathrm{prediction},i}-y_{\mathrm{experimental}}{)}^{2}{)}^{0.5}$$

(22) $$R^{2}\;=\;1-(\sum \;(y_{\mathrm{experimental}}-y_{\mathrm{prediction}}{)}^{2}/\sum \;(y_{\mathrm{experimental}}-y_{\mathrm{average}}{)}^{2})$$

Equation (16) combining Equations (10), (13) and (15) is used for different drug dosages and applied flow rates.

## 3. Results and Discussion

**Figure 4:**
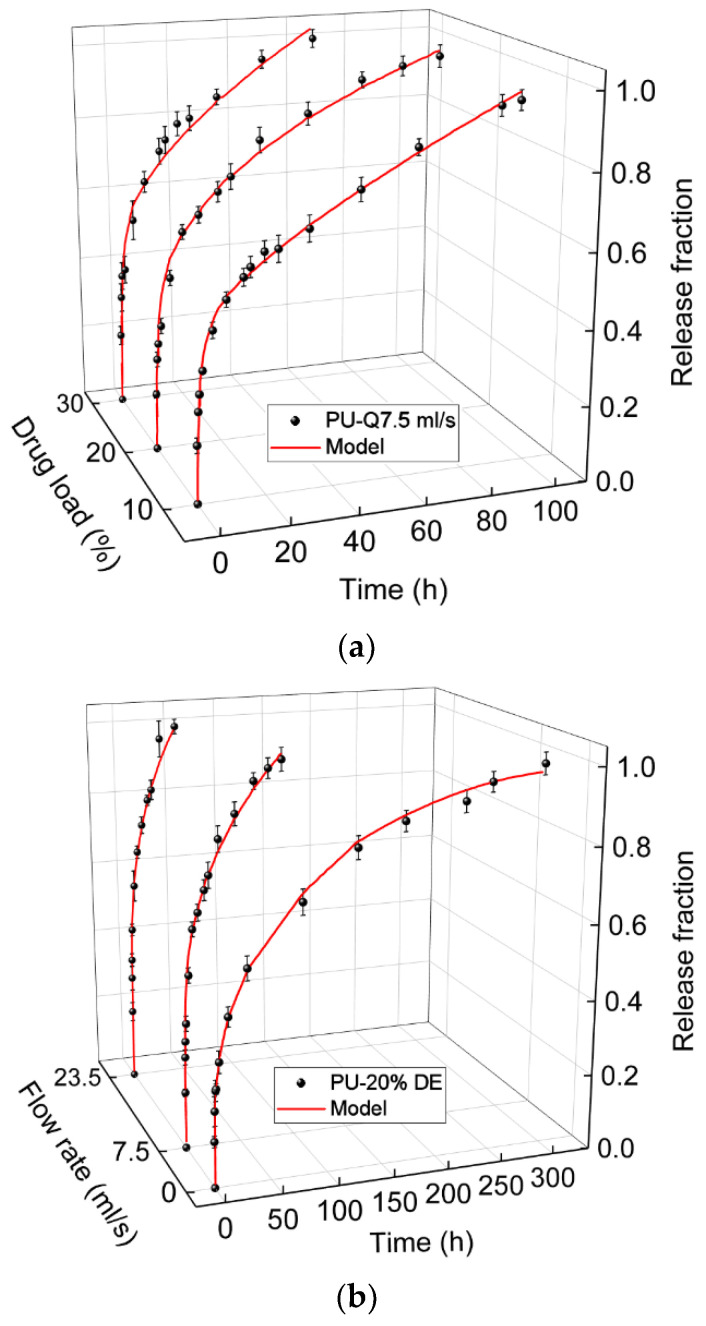
Comparing the calculated values with the Equation (16) and the experimental data of diclofenac release from the polyurethane (PU) matrix, (**a**) for three different drug concentrations; 10%, 20%, and 30% at the flow rate of 7.5 mL/s (**b**) and for the PU with 20% of drug at the flow rates of 0, 7.5, and 23.5 mL/s.

Figure 4 shows the comparison between the experimental and calculated results. Table 1 gives the values related to the percentage of the contribution of the mechanisms associated in the drug release and affecting release kinetics.

### 3.1. Effect of Drug Dosage

**Figure 5:**
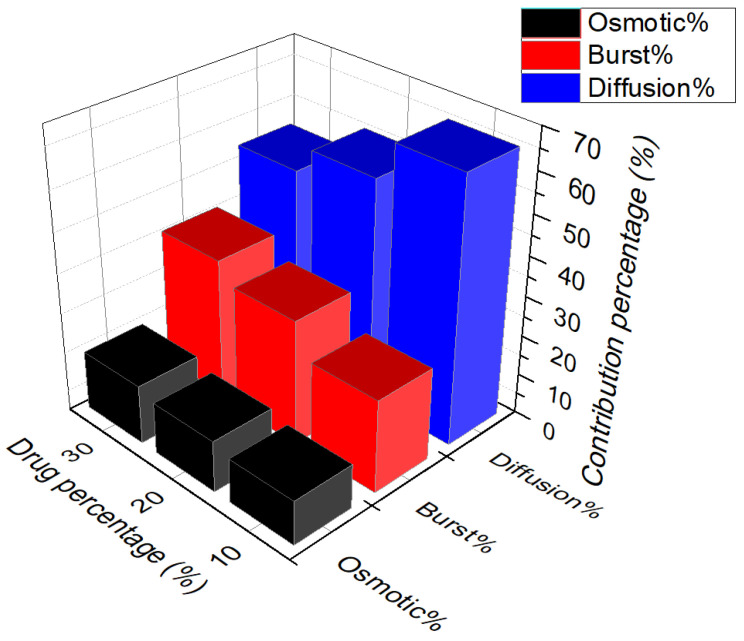
Percentage of the contribution of each mechanism during the drug release from PU films with the three different percentages of the drug at the flow rate of 7.5 mL/s.

Figure 5 represents the contribution-percentage of each three mechanisms at the drug liberation at the flow rate of 7.5 mL/s for the three selected concentrations. One can note that increasing the initial drug percentage results in the increasingly higher burst release. This phenomenon could be explained by the high drug particles delivery, at the initial time of the liberation. This is due to the higher quantity of the drug present on the surface of the samples or in the pores connected to the surface. It is notable that by increasing the ratio of the drug to the total mass of the sample, more drugs can stay at the surface of the sample, especially when the drug is hydrophilic and have the potential to stay at the surface of the samples. Moreover, the kinetics of the release at the burst release is increasing with the drug content. This can be due to the interconnection of the pores containing the drug particles and increasing the feasibility of the release.

Similarly but less significantly, release by the osmosis mechanism is increasing with the quantity of initial drug loaded. One can note that it is the due to the high pores created at the surfaces of the sample, which is at the origin of this phenomenon. Indeed, because of the elevated amount of hydrophilic drug, the permeability of the membrane increase. As the osmosis mechanism in the polymeric films are created due to the high percentage of the water absorbed, increasing the higher hydrophilic drug content increase the water absorption. Therefore, it generates the osmotic pressure from the unwetted core of the sample to the wetted side of the sample. In this case, it is evident that the osmotic pressure exerted for the higher percentage of the drug is higher. 

One can note that it is due to the greater content of the drug especially near to the surface of the samples.

The results show that by increasing the drug percentage the contribution of the diffusion mechanism on the total contribution of the release is decreased. It is evidently because of the higher drug release beforehand by the two aforementioned mechanisms. However, the kinetics of the release by this mechanism, the diffusion coefficient, is increasing by increasing the initial drug loaded. One can note that this is due to the higher mass of the drug remaining in higher drug loaded samples compared to the lower drug loaded samples, even if the percentage liberated from the total percentage is high. Therefore, there is a higher gradient of concentration between the sample and the release medium, which increase the diffusion coefficient of the drug release drug.

**Figure 6:**
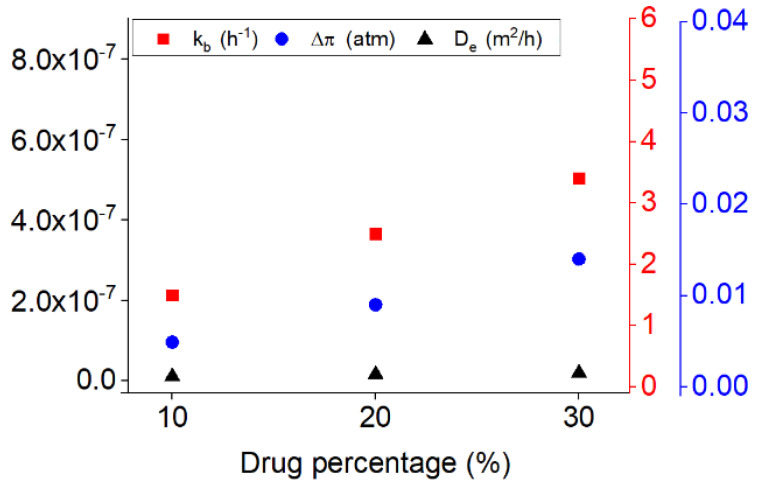
The variation of the release parameters for the drug release from PU films with the three different percentages of the drug (10%, 20%, and 30%) at the flow rate of 7.5 mL/s.

In addition, however, it seems that the diffusion mechanism can be present in all the periods of the release but essentially, it will be the controlling mechanism after the burst and osmosis, where the whole samples become wet and the diffusion can happen in all directions of the sample. Figure 6 shows the variation of the kinetic of burst release, osmotic pressure, and diffusion coefficient versus the initial drug loaded on the samples during the release. It is notable that the kinetics of the release for all the mechanisms by increasing the drug percentages are enhanced. However, it is more evident respectively for the burst release, osmosis and diffusion mechanisms. On can note that generally by increasing the drug dosage, release time is decreasing.

Loading different percentages of the drug affects also the physical properties of the polymer matrix. It is noteworthy that increasing the drug content microscopically and macroscopically creates free volume and space in the polymeric samples, which affect the release behavior especially by the mechanism of osmotic pressure.

**Figure 7:**
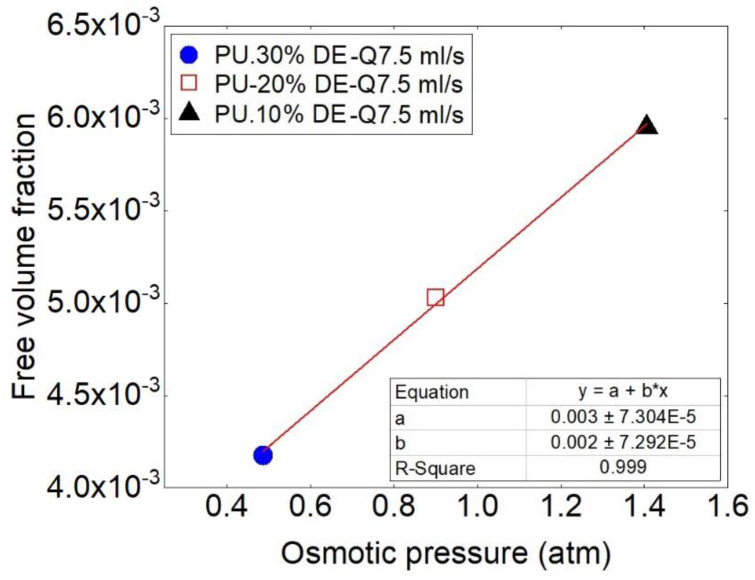
Comparison of the values of free volume fraction and osmotic pressure at the flow rate of 7.5 mL/s for the samples with three different drug concentrations.

Therefore, the importance of the free volume fraction takes consideration. The value of free volume fraction coefficient by using $\left(f_{g}=\sqrt{\frac{B\cdot \Delta \alpha \cdot A}{2.303}}\right)$ for PU + 10% drug, PU + 20% drug and PU+30% drug is calculated respectively, which are determined about 4.18 × 10^−3^, 5.03 × 10^−3^, 5.94 × 10^−3^. Figure 7 shows the comparison of the free volume fraction for different percentages of drug and its effect on the osmotic pressure. By increasing the free volume fraction, osmotic pressure also is increased.

### 3.2. Effect of Flow Rate

**Figure 8:**
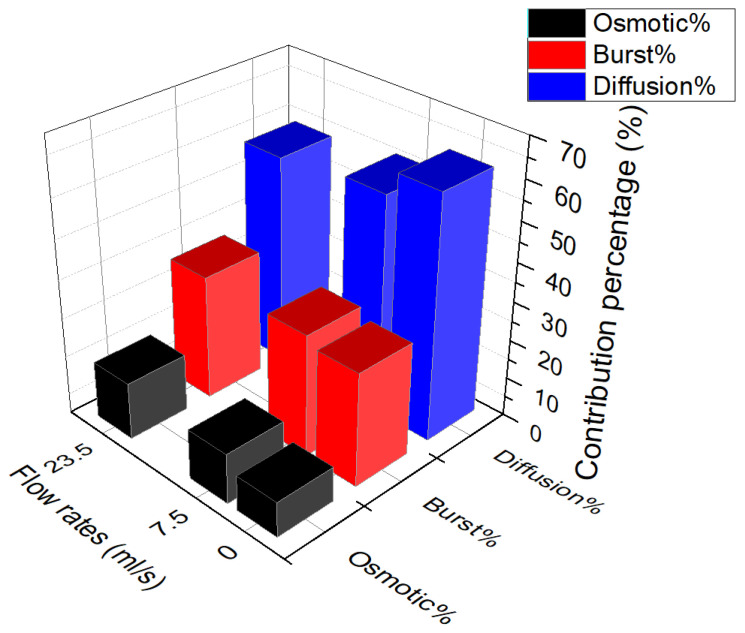
Percentage of the contribution of each mechanism during the drug release from PU films with 20% of the drug at the different flow rates of 0, 7.5 and 23.5 mL/s.

Figure 8 represents the percentages of the contribution of each mechanism during the drug release from PU films with 20% of the drug at the different flow rates of 0, 7.5 and 23.5 mL/s. One can note that by increasing the flow rate the percentage of the burst release is increased.

This increase is less for the two cases of continuous flow rate, but is more visible comparing the results of static and continuous flow. This can be due to the force of the fluid in the continuous state to pluck out the drug particles, but in the static state the dissolution of the drug is needed prior to release. Therefore, it is worth noting that the flow rate mostly influence the kinetics of the burst over influencing the percentage of burst release. It is notable that the effect of the flow rate on the burst release is less notable than the effect of the initial drug loaded. 

Increasing the flow rate also increases the osmotic pressure and more drug is released by this mechanism. Therefore due to the high pressure of the flow for the higher flow rate, high water is absorbed by the polymer and an elevated osmotic pressure is induced in the polymers which results in the drug release. As the polymer and the drug used in this study are hydrophilic, therefore, the amount of the water absorbed is high and lets the solute dissolve in the solvent. In order to counteract the stress caused by the osmotic pressure inside the carrier. The support reduces stress by desorption of the amount of water which can be accompanied with the drug.

**Figure 9:**
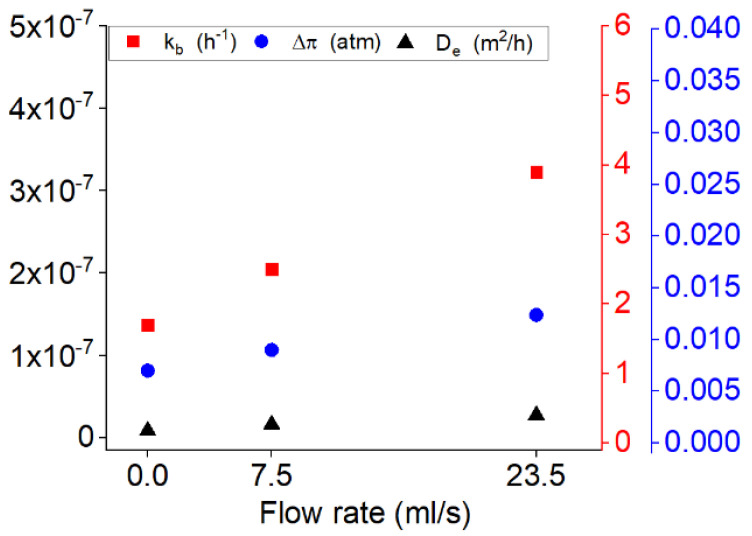
The variation of the release variables for the drug release from PU films with 20% of the drug at different flow rates of 0, 7.5 and 23.5 mL/s.

**Figure 10:**
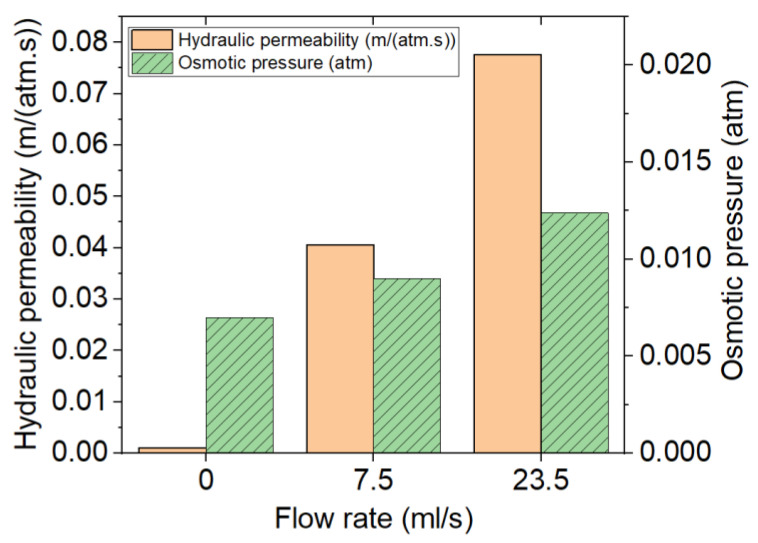
Comparison of the values of permeability and osmotic pressure for samples of PU-20% DE at three different flow rates of 0, 7.5 and 23.5 mL/s.

The effect of the flow rate on the osmotic pressure seems to be the force or pressure applied to the liquid to go through the semipermeable membrane. Therefore, the values related to osmotic pressure seem to increase by increasing the flow rate (shown in Figure 9). In this aim, the permeability of the membrane also remains another determinant parameter in the drug release. Figure 10 shows that the effect of the flow rate is significant on the hydraulic permeability where it is a parameter increasing the percentage of the release by osmotic pressure, and this relation varies linearly.

Another important mechanism which consist a large percentage of the release is diffusion. The lower percentage of the drug released with increasing the flow rate is related to the high amount of drug released beforehand (like as in the effect of concentration) as it is serving a controlling mechanism at the last level. However the kinetics of the release is increased by increasing the flow rate. In particular, by changing the state of the flow to the continuous state gradient of the concentration between the sample and the medium is increased.

Figure 9 shows the variation of the kinetic of the burst release, diffusion coefficient and osmotic pressure along with increasing the flow rate. As shown in Figure 9 the constants related to the burst release, diffusion and osmotic pressure are all increased by increasing the flow rate. This increase is more noticeable respectively for the kinetic of burst, osmotic pressure and diffusion coefficient.

### 3.3. Specific-Model Development and Validation

For analyzing the phenomenon of release, Equation (16) considering three mechanisms of burst, diffusion and osmosis was fitted to the experimental results of the diclofenac release from PU samples at different drug percentages and flow rates. It is shown that the fitting curves with the experimental results in Figure 4 are satisfactory. The parameters resulted from the fittings are summarized in Table 1. The correlation between these values makes it possible to predict the release for the other percentages of drug loaded or other flow rates.

For finding the correlation of the kinetic constants of the occurring mechanisms, (k_b_, D_e_ and Δπ) with the concentration and flow rate, related equation between rate constants with Arrhenius and linear equations were used respectively.

For calculating the rate constants with varying drug concentration, an Arrhenius type equation was used. The applied equation is presented as Equation (23).

(23) $$\mathrm{Ln}\;k=A\left(\frac{1}{C}\right)$$

where k is the constant of the mechanisms, A is the constant of the Arrhenius form equation and C is the initial concentration value.

**Figure 11:**
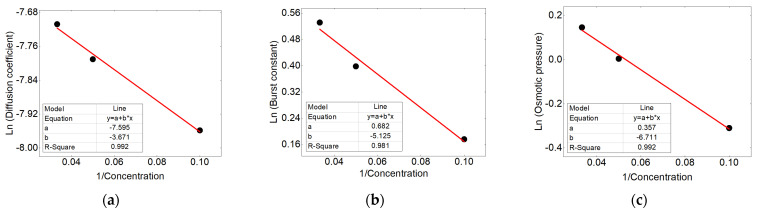
Correlation between (**a**) diffusion coefficient, (**b**) burst constant, (**c**) osmotic pressure in accordance to the concentration adapted to the Arrhenius equation.

The kinetic constants of the mechanisms in accordance to the concentration are traced in the form of the Arrhenius, and the results show good correlation (shown in Figure 11). This correlation were used to calculate the values related to the other loaded percentages.

For calculating the rate constants with varying flow rate, the linear equation was taken into account. Applied equation is presented as Equation (24).

(24) Q=A·k

where k is the constant of the mechanisms, A is the constant of the equation and Q is the flow rate value.

**Figure 12:**
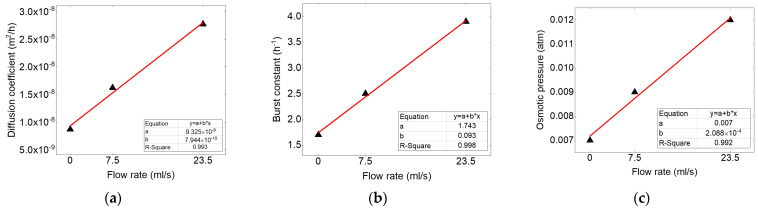
Correlation between (**a**) diffusion coefficient, (**b**) burst constant, (**c**) osmotic pressure in accordance to the flow rate adapted to the linear equation.

The kinetic constants of the mechanisms in accordance to the concentration are traced in the form of the linear, the results shows good correlation (shown in Figure 12). This correlation were used to calculate the values related to the other flow rates.

**Figure 13:**
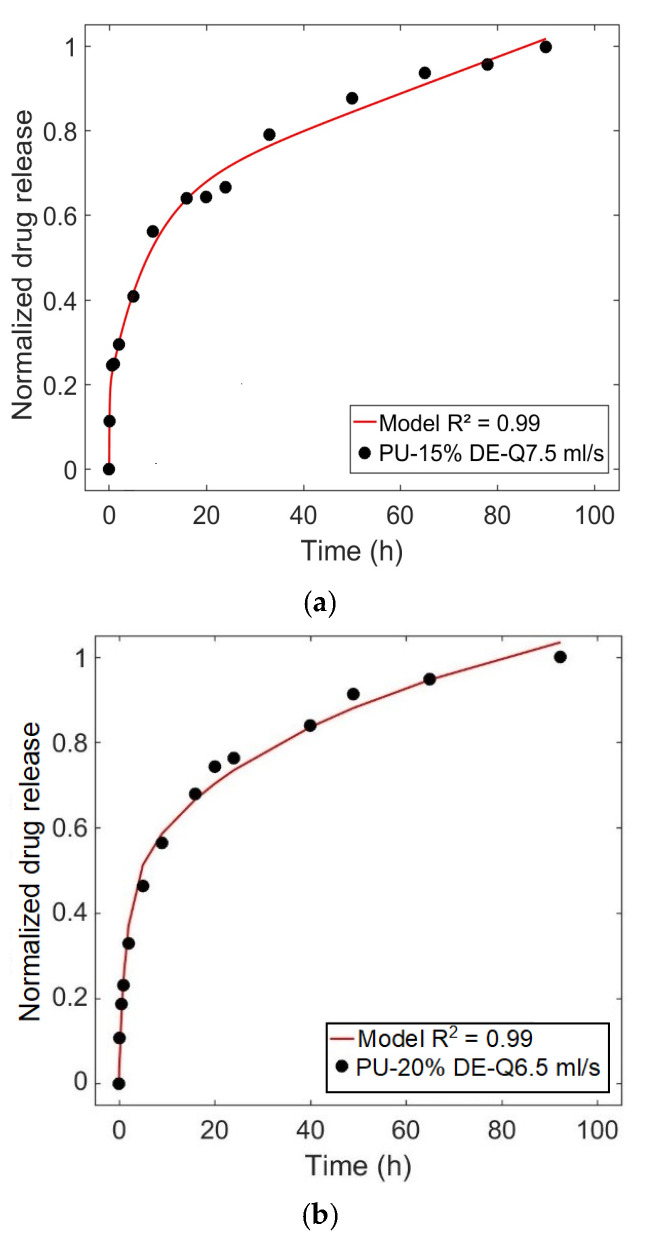
Comparison of the predicted release profile obtained by the model and the experimental data for the case (**a**) PU-15%DE-Q7.5 mL/s, (**b**) PU-20%DE-Q6.5 mL/s.

Figure 13a,b show the predicted drug release profile by the model for the PU matrix loaded with 15% diclofenac at the flow rate of 7.5 mL/s, and 20% diclofenac at the flow rate of 6.5 mL/s compared to the experimental release data. It can be noted that in both cases a good correlation exists between the model and experimental results.

## 4. Perspective

This method can be applied for any kind of drug-delivery agent by considering different effective parameters on the release (such as, temperature, pH, flow rate, initial drug percentage, etc.) in order to optimize the factors in design of these agents adapted to the environment used. However, there are some limitations in this method, such as only the mechanisms that have their own equations can be used (there is a problem using mechanisms such as ion-exchange). This method is considered for the in vitro case, and the in vivo interactions between the drug and tissue components, such as binding-unbinding are not yet taken into consideration.

## 5. Conclusions

A mathematical method to predict the release profile based on physical mechanisms contributing to the release has been developed. In the case studied, three mechanisms for the delivery agent were identified: burst release, diffusion and osmosis. Experimental in vitro data were of the drug release from PU samples with three different concentrations at three different flow rates. The influence of flow rate and concentration on drug release ratios has been highlighted. The results showed that by increasing the drug percentage and flow rate, the contributions of the burst release and osmotics are increased. The effect of the initial concentration was more significant on the burst release and flow rate affected the osmosis mechanism more. It is worth noting that the period of the contribution of a mechanism is also important. Where even though, high proportion of the drug was released by the diffusion mechanism, its contribution decreased with increasing the two parameters. The former can be explained by the fact that diffusion was controlling mechanism almost at the late periods of the release. It is remarkable that the kinetics of the release by three mechanisms of burst, osmotic and diffusion when increasing the two parameters are also increased.

The correlation between the values obtained by the model was helpful in predicting the release behavior at the other range values of that parameter. These parameters have also affected the physical properties, such as free volume fraction and permeability of the materials, which have an influence on the release kinetic. It is shown that the flow rate has linearly affected the osmotic pressure and hydraulic permeability of the samples.

Different parameters can influence the mechanisms contributing to the release. Therefore, the mechanistic models can consider these effects reasonably in order to predict the release behavior and optimize the design of the delivery agent adapted to the environment in use.

## Figures and Tables

**Table 1:** Values related to the percentage of the contribution of the mechanisms associated in the drug release and affecting release kinetic (PU-10%DE-Q7.5 mL/s: polyurethane sample with 10% diclofenac epolamine released at the flow rate of 7.5 mL/s).

Mechanism	PU-10%DE- Q7.5 mL/s	PU-20%DE- Q7.5 mL/s	PU-30%DE- Q7.5 mL/s	PU-20%DE- Q0 mL/s	PU-20%DE- Q23.5 mL/s
Burst (%)	23.2	31.3	36	29	31.9
Osmosis (%)	11.1	12.8	14.7	9	14.6
Diffusion (%)	65.7	55.9	49.3	62	53.5
K_b_ (h^−1^)	1.5	2.5	3.4	1.7	3.9
Δπ (atm)	0.005	0.009	0.014	0.007	0.012
D_e_ (m^2^/h)	1.1 × 10^−8^	1.62 × 10^−8^	1.96 × 10^−8^	8.7 × 10^−9^	2.77 × 10^−8^
RMSE	0.02	0.02	0.03	0.02	0.02
R^2^	0.99	0.98	0.97	0.98	0.99

## Data Availability

The data presented in this study are available on request from the corresponding author.
